# A new method to infer higher-order spike correlations from membrane potentials

**DOI:** 10.1007/s10827-013-0446-8

**Published:** 2013-03-10

**Authors:** Imke C. G. Reimer, Benjamin Staude, Clemens Boucsein, Stefan Rotter

**Affiliations:** Bernstein Center Freiburg and Faculty of Biology, University of Freiburg, Freiburg, Germany

**Keywords:** Shot noise process, Intracellular recording, Subthreshold activity, Presynaptic population, Correlated neuronal groups

## Abstract

What is the role of higher-order spike correlations for neuronal information processing? Common data analysis methods to address this question are devised for the application to spike recordings from multiple single neurons. Here, we present a new method which evaluates the subthreshold membrane potential fluctuations of one neuron, and infers higher-order correlations among the neurons that constitute its presynaptic population. This has two important advantages: Very large populations of up to several thousands of neurons can be studied, and the spike sorting is obsolete. Moreover, this new approach truly emphasizes the functional aspects of higher-order statistics, since we infer exactly those correlations which are seen by a neuron. Our approach is to represent the subthreshold membrane potential fluctuations as presynaptic activity filtered with a fixed kernel, as it would be the case for a leaky integrator neuron model. This allows us to adapt the recently proposed method CuBIC (cumulant based inference of higher-order correlations from the population spike count; Staude et al., J Comput Neurosci 29(1–2):327–350, 2010c) with which the maximal order of correlation can be inferred. By numerical simulation we show that our new method is reasonably sensitive to weak higher-order correlations, and that only short stretches of membrane potential are required for their reliable inference. Finally, we demonstrate its remarkable robustness against violations of the simplifying assumptions made for its construction, and discuss how it can be employed to analyze *in vivo* intracellular recordings of membrane potentials.

## Introduction

Neurons can be sensitive to synchronized input (e.g. Abeles 1982; Rudolph and Destexhe 2003; Cardin et al. 2010; Rossant et al. 2011; Hong et al. 2012). In particular, the output firing rate and subthreshold membrane potential fluctuations of a neuron can be affected not only by the pairwise correlations of the presynaptic spikes but also by the higher-order structure of the input (Bohte et al. 2000; Kuhn et al. 2003; Benucci et al. 2007; Pelko and van Rossum 2011).

Higher-order spike correlations *in vitro* and *in vivo* are commonly studied in parallel spike trains which have been recorded extracellularly (Martignon et al. 2000; Shlens et al. 2006; Schneidman et al. 2006; Tang et al. 2008; Montani et al. 2009; Shlens et al. 2009; Ohiorhenuan et al. 2010; Ganmor et al. 2011a, b; Ohiorhenuan and Victor 2011; Yu et al. 2011; Shimazaki et al. 2012). This approach has three obvious disadvantages: Firstly, the sampled neuronal population is restricted and biased in various ways. The sample size is constrained by the number of electrodes that can be employed simultaneously. However, pairwise spike correlations are rather weak (e.g. Kohn and Smith 2005; Renart et al. 2010) and, hence, a really large neuronal population must be considered to reliably judge on the existence of higher-order correlations. Moreover, the recorded neuronal pool is biased towards large and frequently spiking neurons, whereas the majority of cortical neurons fires sparsely (Barth and Poulet 2012). Secondly, most methods to infer higher-order correlations require single unit activity and, hence, spike sorting is necessary (for exceptions see Ehm et al. 2007; Staude et al. 2010b, c). Not only is this a troublesome procedure but also is the measurement of neuronal correlations particularly sensitive to spike sorting errors (Gerstein 2000; Bar-Gad et al. 2001; Pazienti and Grün 2006; Cohen and Kohn 2011; Ventura and Gerkin 2012). Thirdly, even if higher-order correlations are statistically significant, this does not imply that they are “seen” by other neurons at the next processing stage. In fact, higher-order events may be a side-effect of the network structure and not a functional feature.

**Fig. 1:**
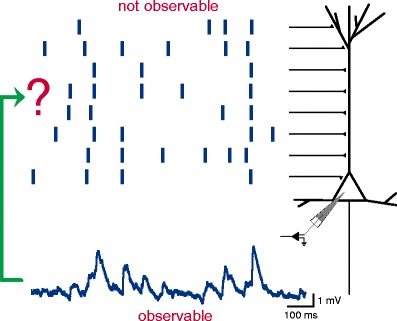
Relating subthreshold membrane potential fluctuations of a neuron and its presynaptic input spike trains. A neuron receives spikes which have been elicited by many presynaptic neurons (raster plot at *top*). While this activity is not observable, intracellular recordings allow to observe the subthreshold membrane potential fluctuations of a neuron (*bottom*). This signal contains information about the input spiking dynamics. For instance, coincident spikes give rise to deflections in the membrane potential. Thus, analysis of this signal may reveal higher-order spike correlations in the input population (*arrow*). Membrane potential trace has been recorded intracellularly *in vivo* in rat primary visual cortex

These problems, however, are resolved if one considers the neuron as an “electrode” which samples from its own presynaptic population (see Fig. 1). A neuron receives the input of thousands of other cells which, at least in cortical networks, are often located far away (Boucsein et al. 2011). Hence, the neuron actually records the activity of a very large population which is neither restricted to a local volume with cells of high firing rates nor observable by us. However, this presynaptic, possibly correlated, spike activity is encoded in the membrane potential of the postsynaptic neuron which is observable. Thus, analysis of the subthreshold membrane potential fluctuations can, in principle, reveal the higher-order spike correlations “seen” by this neuron.

So far, this ansatz to infer cooperative dynamics has only rarely been implemented. Only approaches are available that either calculate the mean presynaptic pairwise spike correlations from conductance fluctuations (Rudolph and Destexhe 2006), or that yield an order-of-magnitude estimate of the number of presynaptic neurons participating in a synchronous event from subthresold membrane potentials (Léger et al. 2005; DeWeese and Zador 2006).

Here, we present a new method to infer higher-order spike correlations from filtered spike activity, in particular, from subthreshold membrane potential fluctuations. Similarly to Rudolph and Destexhe (2006), and in line with the common leaky integrator neuron model, we conceive the subthreshold activity as presynaptic correlated spike trains subject to spatio-temporal summation with a fixed filter kernel. This representation allows to adapt a recently proposed method based on binned population spike activity, called CuBIC (cumulant based inference of higher-order correlations by Staude et al. 2010a). This is possible since counting spikes is identical to filtering with a rectangular kernel and evaluating at discrete multiples of the bandwidth. By resorting to the cumulants of the population spike count, or subthreshold activity, respectively, a lower bound for the maximal order of correlation can be inferred by a sequence of hypothesis tests (Section 2). However, as a result of overlapping filter kernels sample data points are not independent of each other and a procedure to correct for the implied redundancy has to be implemented (see Section 3). On surrogate data we demonstrate that our new method CuBICm (CuBIC for membrane potentials) is very sensitive to weak higher-order correlations and requires only small stretches of membrane potential to give good estimates (Section 4). Finally, we show that CuBICm is robust against violations of the underlying model assumptions and discuss its applicability to real neuronal data (Sections 5 and 6).

## CuBICm: adaptation of CuBIC

Initially, we describe our model for the subthreshold activity of a neuron before we explain how higher-order correlations can be inferred from experimental data. The relation of CuBICm and CuBIC will be clarified in Section 2.1.2.

### Model

The Model comprises two components: the presynaptic spike activity with higher-order correlations and the postsynaptic subthreshold activity.

#### Presynaptic spike activity and higher-order correlations

**Fig. 2:**
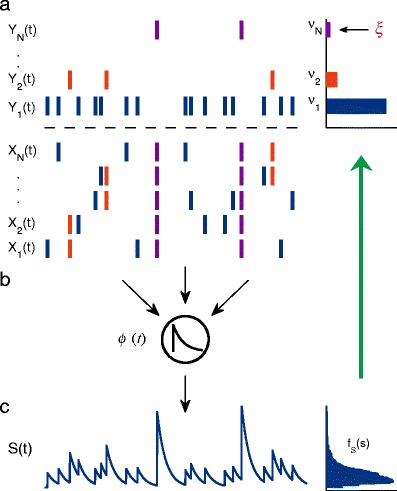
Model and measurement. **a** Presynaptic spike activity. The sum activity $\sum X_{i}(t)$ is described as a compound Poisson process $Z(t)=\sum n Y_{n}(t)$, where $Y_{n}(t)$ are independent Poisson processes with intensity $\nu _{n}$. Each process $Y_{n}(t)$ represents the synchronized activity of *n* presynaptic neurons encoded by the same color. **b** Neuronal integration. The biological procedure is modeled as convolution with a fixed kernel $\phi (t)$. **c** Postsynaptic subthreshold activity. Filtering of the presynaptic spike activity yields a shot noise process $S(t)=(Z \ast \phi )(t)$ with amplitude distribution $f_{S}(s)$. From the cumulants of this distribution the maximal order of correlation $\xi $ can be inferred via CuBICm (indicated by *green arrow*)

Figure 2a (bottom) shows the spike trains $X_{1}(t),X_{2}(t), \ldots, X_{N}(t)$ of a population of *N* presynaptic neurons. As is highlighted by red bars, spikes of different neurons can occur at the same time. We model the summed spike activity of the population, $\sum _{i} X_{i}(t)$, as a compound Poisson process (CPP)

1 $$ Z(t)=\sum\limits_{n}{n Y_{n}(t)}. $$

Here, the component processes $Y_{n}(t)'s$ are independent stationary Poisson processes with intensity $\nu_{n}$ (Fig. 2a, top). Each process $Y_{n}(t)$ represents the times of synchronized activity of *n* neurons as is indicated by corresponding colors. Thus, if $\nu_{n}>0$ for some *n*, the spike activity of the presynaptic neurons exhibits correlations at least of order *n*. Accordingly, we refer to $(\nu_{1},\nu _{2},\ldots ,\nu _{N})$ as the correlation structure and call $\xi =\max \{n|\nu _{n}>0\}$ the maximal order of correlation. In the example of Fig. 2a $\nu_{N}>0$ and, thus, $\xi =N$.

The representation of the summed presynaptic spike activity as a composition of *N* independent processes $Y_{n}(t)'s$ is crucial for the derivation of our method (see following Section). Moreover, the CPP allows the description of a broad range of spiking neuronal populations (see Ehm et al. 2007; Brette 2009; Staude et al. 2010a, b, c). In particular, it is not restricted to a homogeneous pool and neurons can exhibit different firing rates and pairwise correlations. For simplicity, the reader can think of the single cell processes given by the CPP as stationary Poisson processes. We will get back to this issue in Section 5.1.2 (Poisson) and Section 6 (non-stationarity).

#### Postsynaptic subthreshold activity

##### Model

We consider the postsynaptic neuron as a point-neuron where the synaptic integration is modeled as equally weighting, summing and then filtering the input spikes (Fig. 2b). More precisely, we represent the subthreshold activity *S* of a neuron as presynaptic population spike activity *Z* Eq. (1) convolved with a fixed kernel $\phi $ (Fig. 2c):

2 $$ S(t)=(Z \ast \phi)(t) $$

Note, that the assumption of a fixed kernel means, in particular, to have either a positively or a negatively signed filter kernel and, hence, either excitatory or inhibitory input only.

1. *Example: Membrane potential fluctuations* If *S* represents the subthreshold membrane potential fluctuations a reasonable choice for the filter kernel $\phi $ is an exponential function
  3 $$ \phi(t)=A \exp\left(-\frac{t}{\tau}\right) $$
  where *A* is the amplitude of the postsynaptic potential due to one presynaptic spike and $\tau $ is the membrane time constant. In fact, this model is equivalent to the well-known and frequently employed representation of a neuron as a leaky integrate and fire neuron with pulse-like synaptic current input. Its subthreshold activity is more often described by the differential equation
  4 $$ \frac{\mathrm{d}}{\mathrm{d}t}U(t)= \frac{- [U(t)-U_{r}]}{\tau}-\frac{I(t)}{C} $$
  with membrane potential $U(t)$ (i.e., $U=S$), current input $I(t)$, membrane capacitance *C*, and resting membrane potential $U_{r}$ (Tuckwell 1988). Equation (3) is a solution for $U_{r}=0$ and $I(t)=AC \delta (t)$ where $\delta (t)$ is the Dirac delta function.
2. *Example: Population spike count* The original method CuBIC to infer higher-order correlations operates on the population spike count where this spike count is obtained by binning the population spike activity. Binning can be described as filtering with a rectangular kernel of length *h* evaluated at discrete multiples of the bin size *h*. Hence, the population spike count can be expressed in terms of Eq. (2).

##### Cumulants

The estimation of the maximal order of correlation in the presynaptic spike activity will be based on three characteristics of the distribution $f_{S}(s)$ of the postsynaptic subthreshold activity *S* (Fig. 2c, right): the mean postsynaptic activity, its variance and a measure similar to the skewness of the distribution. Statistically this corresponds to the first, second and third cumulant. For our model where *Z* is a CPP (see Eq. (1)), the m-th cumulant of *S* and, hence, the first ($m=1$), second ($m=2$) and third ($m=3$) cumulant, is given by

5 $$ \kappa_{m}[S]=\kappa_{m}\left[\sum\limits_{n} (n Y_{n} \ast\phi)\right] $$

6 $$ =\sum\limits_{n} n^{m} \kappa_{m}[Y_{n} \ast \phi] $$

7 $$ =\sum\limits_{n} n^{m} \nu_{n} \int \phi^{m}. $$

From line Eq. (5–6) it has been exploited that the processes $Y_{n}'s$ are independent and hence, the additivity of cumulants applies. Line Eq. (6) is a result of the fact that $Y_{n}$ is a Poisson process (Yue and Hashino 2001). As line Eq. (6) shows, the cumulants of *S* contain all informations about the correlation structure of the presynaptic spike activity. In the following section we explain how this information can be used to infer a lower confidence bound for the maximal order of correlation.

### Measurement

As depicted in Fig. 2, higher-order events in the input give rise to large deflections in the subthreshold activity *S* and, thus, put weight to the tail of the distribution $f_{S}(s)$ and increase the skewness of $f_{S}(s)$. This can be characterized by the first three cumulants of *S*. More precisely, under the assumption of our model (Section 2.1) the cumulants of *S* are just a weighted sum of the rates of presynaptic synchronized activity of all orders (cf. Eq. (7)). Also, we know that given the first and second cumulant, the third cumulant cannot exceed a certain value if there are correlations only up to order *k*, i.e. $\nu_{n}=0$ for $n>k$ (cf. Staude et al. 2010c).

#### Inference of a lower bound for the maximal order of correlation

As in the original CuBIC we construct a statistical test based on this feature. The null hypothesis is

$$\mathrm{H}^{k}_{0}: \text{ `the first three cumulants are consistent with correlations not exceeding order k'.}$$

Thus, rejection of this null hypothesis tells us that there are at least correlations of order *k*. Applying this test successively for increasing order of correlation *k* then gives a lower confidence limit for the maximal order of correlations by

$$\hat{\xi}=\min\left\{k{\kern2pt} | {\kern2pt} \mathrm{H}^{k}_{0} \text{ cannot be rejected}\right\}. $$

In order to calculate a p-value $p_{k}$ associated with $\mathrm {H}^{k}_{0}$ one has to proceed as follows: Initially, we need to measure the first three sample cumulants $k_{1}, k_{2}$ and $k_{3}$ of the subthreshold activity. Then we must determine an upper bound for the third cumulant under $\mathrm {H}^{k}_{0}$, denoted $\kappa _{3,k}^{*}$. As we assume the CPP model, all cumulants have to fulfill Eq. (7). Under $\mathrm {H}^{k}_{0}$ there are no correlations of order greater than *k* and, hence, $\nu _{n}=0$ for $n>k$. Taken together, $\kappa _{3,k}^{*}$ is obtained by solving the maximization problem

8 $$ \kappa_{3,k}^{*}=\max\limits_{(\nu_{1},\nu_{2},\ldots,\nu_{k},0,\ldots,0)} \sum\limits_{n=1}^{k} n^{3} \nu_{n} \int \phi^{3}$$

9 $$ \text{s.t.}~~ k_{1} =\sum\limits_{n=1}^{k} n \nu_{n} \int \phi$$

10 $$ k_{2} =\sum\limits_{n=1}^{k} n^{2} \nu_{n} \int \phi^{2}$$

Here, we presupposed $\int \phi ^{3}>0$. The case $\int \phi ^{3}<0$ can be treated by finding the minimum instead of the maximum. Following the arguments in Staude et al. (2010b), the optimization problem can be solved analytically and is achieved for a correlation structure with non-zero entries at $n=1$ and $n=k$ only, i.e. $(\nu _{1}^{*},0,0,\nu _{k}^{*},0,\ldots )$ (see Appendix A).

Finally, the p-value $p_{k}$ of the associated hypothesis test can be calculated via noting that the third sample cumulant is approximately normally distributed under the null hypothesis with mean $\kappa _{3,k}^{*}$ and a variance $\sigma _{k}^{{*}^{2}}$ which incorporates the m-th cumulants $\kappa _{m,k}^{*}$ up to order six (see Appendix B):

11 $$ p_{k} = 1- \Phi\left(\left(k_{3}-\kappa_{3,k}^{*}\right) / \sigma_{k}^{*}\right) $$

where $\Phi $ denotes the cumulative distribution function of the standard normal distribution.

#### Requirements

The kernel $\phi $ and its parameters need to be known in order to determine the upper bound for the third cumulant under $\mathrm {H}^{3,k}_{0}$ (see Eqs. (8–10)) and, thus, to infer the order of correlation $\xi $ of the presynaptic spike activity. However, we do not need to know the number of input neurons *N*. Furthermore, the CPP model allows for *any* correlation structure in a neuronal population of heterogeneous spike statistics and is not restricted to e.g. a binomial-like distribution of higher-order correlations in a homogeneous pool as in Rudolph and Destexhe (2006). If a membrane voltage trace is analyzed, an estimate of the resting membrane potential $U_{r}$ is required and has to be subtracted from the signal (cf. Eqs. (3) and (4)). Here, we assume the corresponding case of $U_{r}=0$.

## Correction for correlated samples

With the new method at hand we can consider a first example.

### Example

Initially we treat the simple case of independent presynaptic processes and ask how CuBICm performs. In so doing, we modeled the postsynaptic activity by filtering the presynaptic spike trains with an exponential kernel $\phi (t)$ (see Eq. (3)). If not stated otherwise, we set $A=1$ and simulated the shot noise process $S(\Delta t)$ via exact integration (Rotter and Diesmann 1999) with a time resolution of $ \Delta t= 0.05 \,\mathrm {ms}$ which corresponds to a sample rate of 20 kHz. Moreover, we avoided onset transients by the use of a warm up time of 1 s.

**Fig. 3:**
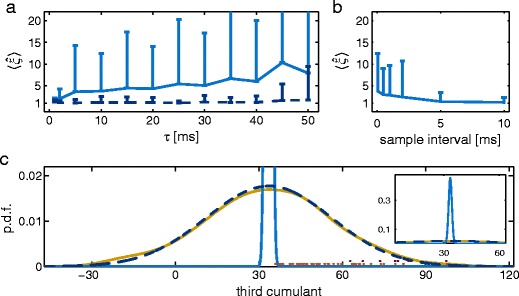
Impact of correlated samples and associated correction. **a** Inferred maximal order of correlation in dependence of the filter time constant $\tau $ for a shot noise process with presynaptic activity consisting of $N=200$ independent Poisson processes with rate $\lambda =10$ spikes/s each. Average results over 200 simulations for 50 s each are shown for CuBICm without correction for correlated samples (*light blue*) and CuBICm with correction (*dark blue, dashed line*). **b** Inferred $\xi $ for different sample intervals for the same shot noise processes as in (a) with $\tau =10\,\mathrm {ms}$. Simulation time has been adjusted to keep the sample size fixed. **c** Distribution of third cumulant of data in (a) with $\tau =10\,\mathrm {ms}$. Kernel density estimate of third sample cumulant (*yellow*) is shown in comparison with the *mean* distribution assumed under $H_{0}^{1}$ for CuBICm without correction (*light blue*) and CuBICm with correction (*dark blue, dashed line*). Additionally, dots indicate the cumulants of the data sets for which $H_{0}^{1}$ has been rejected by the use of CuBICm without correction (*light red*) and CuBICm with correction (*dark red*) which corresponds to 22.5 % and 3.5 % of all data sets, respectively. *Inset* presents same figure but with different axes limits. *Error bars* in *a–b* depict standard deviation. A significance level of $\alpha =0.05$ has been used

As can be seen in Fig. 3a (light blue), it can happen that the actual order of correlation (i.e., $\xi =1$) is overestimated by CuBICm. More precisely, this bias becomes more prominent with longer time constants of the kernel. Therefore, we suggest that this is due to correlated samples: Recall that the sample cumulants are unbiased estimators of the true cumulants only if the samples are independent. In contrast, filtering spike trains with a kernel which has long time constants may imply a large memory of previous activity.

We tested our hypothesis by generating data where only every i-th data point is used for the estimation. In doing so, we adapted the simulation time accordingly to keep the sample size *L* fixed and hence, ensure that the results are really comparable. One can see in Fig. 3b that with increasing sampling interval the mean estimated order of correlation goes down to the true order of correlation. Hence, misestimation of $\xi $ is due to an application of CuBICm to samples which are not independent.

### Correction

The above approach of skipping samples requires increased stretches of signals (e.g. membrane potentials) and, therefore, it is not applicable to a given sample of experimental data. Also, adjusting the experimental design accordingly is not practicable. For instance, for the data set in Fig. 3b with $\tau =10$ ms a downsampling with an interval of 10 ms was required to obtain an unbiased estimate of the order of correlation. For an experimental sample rate of 20 kHz this, however, corresponds to almost 3 h of recording time to get the same sample size as for 50 s. And for larger membrane time constants the recording time has to be increased accordingly. Thus, an alternative is required to overcome the problem of correlated samples. In order to find a solution we need to better understand what is going wrong: As CuBICm is based on estimates of the first three sample cumulants and the variance of the third sample cumulant under the null hypothesis, the impact of correlated samples must be visible in these quantities. We observed that the first three cumulants are estimated quite well (not shown). In contrast, the standard deviation of the third sample cumulant is the stronger underestimated the longer the time constants are. That is, using the hypothesis test we assume a normal distribution of the third sample cumulant which is too narrow as compared to the actual one (light blue and yellow line, respectively, in Fig. 3c for $\tau =10\,\mathrm {ms}$). Therefore, the probability that the third sample cumulant is bigger than assumed under the null hypothesis is much higher than it should be from the chosen significance level $\alpha $ (i.e., $P(p_{k} \leq \alpha )>\alpha $). Hence, we tend to falsely reject the null hypothesis (cf. the light red dots in Fig. 3c and, as a result, we overestimate the maximal order of correlation.

In order to correct the standard deviation of the third sample cumulant we chose the following approach: We estimate the ratio of misestimation of the standard deviation as a corrective factor $f_{c}$ for independent processes and use $f_{c} \cdot \sigma _{k}^{*}$ instead of the theoretical standard deviation $\sigma _{k}^{*}$ for the hypothesis test associated with $\mathrm {H}^{k}_{0}$. More precisely, we approximate the rate of independent spike activity by $\lambda = {k_1} / {\int \phi(t)},dt$ (cf. Eq. (9)) where $k_{1}$ denotes the first sample cumulant and use this to generate various times (here: 20 times) a Poisson process with this rate $\lambda $. Thereafter, we filter these spike trains with the same kernel as had been assumed for the original process. Based on the resulting shot noise processes we then calculate $f_{c}= {\rm std} (k_{3})/\sigma _{1}^{*}$ where ${\rm std} (k_{3})$ denotes the empirical standard deviation of the third sample cumulants and $\sigma _{1}^{*}$ is the theoretical standard deviation under $\mathrm {H}^{1}_{0}$. Hence, we suppose that the relative bias made for $\sigma _{1}^{*}$ is the same as for $\sigma _{k}^{*}$ with $k>1$. But this procedure implies no assumptions additional to the ones already made.

Figure 3c illustrates that the average corrected distribution of the third sample cumulant (dark blue dashed line) fits the empirical one (yellow line) quite well. As a result, the actual order of correlation is not overestimated by this method (Fig. 3a, dark blue dashed line).

In the following chapters we will always use CuBICm which has this corrective procedure implemented. Moreover, we will focus on the exponential filter kernel and the interpretation of the subthreshold activity as membrane potential fluctuations. If not stated otherwise, simulations were performed with *Matlab* and the code for CuBICm will be made available at www.apst.spiketrain-analysis.org/Analysis_Software.

## Sensitivity

The previous section showed how we can avoid to get more false positives than allowed by the significance level of the hypothesis test. Here we illustrate via extensive simulation studies that this adapted version of CuBICm is sensitive and able to detect weak higher-order correlations present in the presynaptic spike activity.

### Data sets

**Fig. 4:**
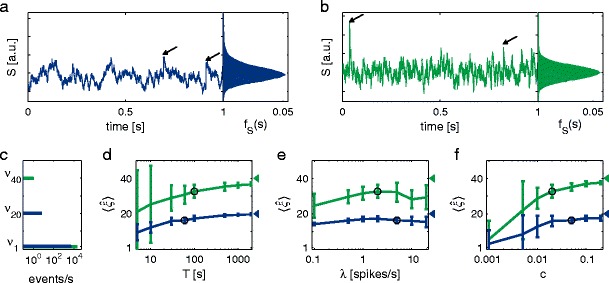
Subthreshold activity and corresponding estimated maximal order of correlation in dependence of various parameters. Presynaptic activity is mimicked as *N* Poisson processes, where a subpopulation of $N_{c}$ is correlated with pairwise correlation coefficient *c*. Each presynaptic neuron fires at rate $\lambda $. previous sectione performed for time *T*, and each data set filtered with an exponential kernel with time constant $\tau $. *Blue*: Data set with default parameters $N=1000, N_{c}=100, c=0.05, \lambda =5\,\mathrm {spikes/s}, \tau =20\,\mathrm {ms}, T=60\,\mathrm {s}$. *Green*: Data set with default parameters $N=10000, N_{c}=200, c=0.02, \lambda =2\,\mathrm {spikes/s}, \tau =5\,\mathrm {ms}, T=100\,\mathrm {s}$. **a**, **b** Sample of 2 s of subthreshold activity *S* and its distribution for the whole data trace. *Arrows* indicate time stamps of higher-order events in the presynaptic spike activity. **c** Correlation structure. **d**, **e**, **f** Estimated maximal order of correlation averaged over 50 simulations. *Error bars* represent standard deviation. *Black circles* depict results for default parameter settings and *triangle mark* true maximal order of correlation

The number of presynaptic neurons, the rate of spiking, the strength of correlation and the membrane time constant depend on various factors like brain area, animal, neuron type, experimental preparation and stimulus. Here we restrict ourselves to two data sets whose parameters we will vary: First, a small presynaptic population of $N=1000$ neurons where a subpopulation of $N_{c}=100$ neurons exhibit synchronized activity of order 20 with a strength corresponding to a pairwise correlation coefficient of $c=0.05$ (cf. correlation structure in Fig. 4c, blue bars). Each presynaptic spike train has been realized by a Poisson process with $\lambda =5$ spikes/s for $T=60$ s. These processes were convolved with an exponential kernel with time constant $\tau =20\,\mathrm {ms}$ to simulate the subthreshold postsynaptic membrane potential (Fig. 4a). The second example is a bigger and sparser spiking presynaptic population with $N=10000, N_{c}=200$ and $\lambda =2$ spikes/s for $T=100$ s. Simultaneous spikes occur across 40 neurons with $c=0.02$ (Fig. 4a, green bars). The postsynaptic neuron integrates the incoming spikes with a time constant of $\tau =5\,\mathrm {ms}$ (Fig. 4b).

### Inferred maximal order of correlation

As Fig. 4a, b show, higher-order correlations are hardly visible in the postsynaptic activity of both examples (see arrows, left). Also the distributions of the full data stretches are not obviously skewed which would indicate the existence of presynaptic multi-neuron events (Fig. 4a, b, right).

However, in both cases using the correct exponential kernel CuBICm can infer a maximal order of correlation $\hat {\xi }$ which is close to the true value (compare black circles with triangles in Fig. 4d). On average, $\hat {\xi }$ becomes a bit smaller when the simulation time, i.e. the sample size, is decreased (blue and green lines, corresponding to Fig. 4a and b, respectively). But even for data stretches of only 5 s CuBICm still detects higher-order correlations (Fig. 4d).

Changing the firing rate of the presynaptic neurons between 0.1 and 20 spikes/s has a less pronounced effect on the test performance than changing the simulation time and no clear trend is visible (see Fig. 4e). In contrast, the inferred maximal order of correlation strongly depends on the strength of cooperative activity as measured by the pairwise correlation coefficient *c* (Fig. 4f). While for $c=0.1$ the actual order is correctly inferred, reasonable results are obtained also for very weak correlations of $c=0.01$. This is even more surprising given that correlations occur only within a small subpopulation of the input neurons.

**Fig. 5:**
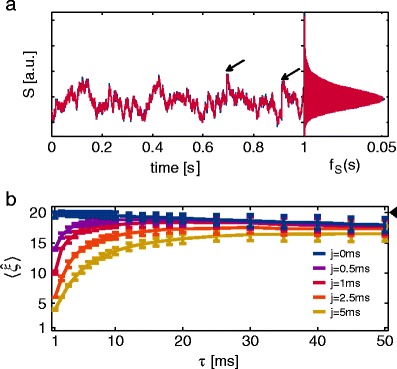
Mean inferred maximal order of correlations for imprecise coincidences. Presynaptic activity as in Fig. 4b. All spike times of the presynaptic activity have been jittered according to a uniform distribution with support $[-j,+j]$. **a** Same shot noise process as in Fig. 4b (*blue*) and the same data with jitter $j=1\,\mathrm {ms}$ (*red*). **b** Mean estimated maximal order of correlation in dependence of time constant $\tau $ for various degrees of jitter (*blue*: $j=0\,\mathrm {ms}$, *purple*: $j=0.5\,\mathrm {ms}$, *red*: $j=1\,\mathrm {ms}$, *orange*: $j=2.5\,\mathrm {ms}$, *yellow*: $j=5\,\mathrm {ms}$). *Error bars* depict one time standard deviation. Simulation time is 500 s

The test performance does not depend on the amplitude *A* of the exponential filter kernel (not shown). However, the smaller the time constant $\tau $ the better are the results given the same sample size which is shown in Fig. 5 (blue line) for the data set of $N=1000$ neurons.

## Robustness

As we illustrated in the previous section CuBICm can detect weak higher-order correlations in short stretches of subthreshold activity. However, our proposed method to estimate higher-order correlations “seen” by a neuron rests on various assumptions. Here, we will investigate its robustness against violations of these assumptions. In doing so, we will mainly treat aspects which are specific to this adapted version of CuBIC. Again we simulate and analyze signals by the use of an exponential filter kernel unless stated otherwise.

### Presynaptic spike activity

We assume the compound Poisson process (CPP) as a model for the presynaptic spike activity which implies precise coincidences and Poissonian spiking of single neurons.

#### Imprecise coincidences

Synchronized spiking occurs on a time scale of milliseconds (see e.g. Riehle et al. 1997; Kohn and Smith 2005, for pairwise spike time correlations). In order to mimic this scenario we added to all spike times a random variable with uniform distribution on $[-j,+j]$. As can be seen in Fig. 5a, a jitter of $j\pm 1\,\mathrm {ms}$ hardly affects the subthreshold activity and its distribution for our example Fig. 4b with $\tau =20\,\mathrm {ms}$. In accordance with this observation, Fig. 5b demonstrates that the order of correlation is well estimated if the time constant is sufficiently bigger than the degree of imprecision. Otherwise, $\xi $ tends to be underestimated. The membrane time constant of cortical neurons has been shown to be a crucial determinant for the length of the window for temporal sensitivity (Cardin et al. 2010). Along these lines, our results do not constitute a weakness of our method but rather suggest how higher-order spike correlations are processed by different single neurons in terms of their subthreshold activity.

#### Non-poissonian spiking

**Fig. 6:**
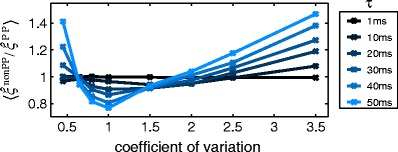
Impact of non-Poissonian spiking on the inference of higher-order correlations. Average results for presynaptic processes with lognormal inter-spike intervals, $\hat {\xi }^{nonPP}$, in relation to estimates, $\hat {\xi }^{PP}$, for presynaptic Poisson processes are depicted. Non-Poissonian and Poisson processes have the same correlation structure. Lognormal processes with different coefficient of variations of their inter-spike interval distribution are considered. Statistics of presynaptic population are identical to the blue data set in Fig. 7a. An exponential filter kernel with amplitude $A=1$ and different time constants $\tau $ between 1 ms (*black*) and 50 ms (*light blue*) has been used. Simulation time is $T=100$ s

**Fig. 7:**
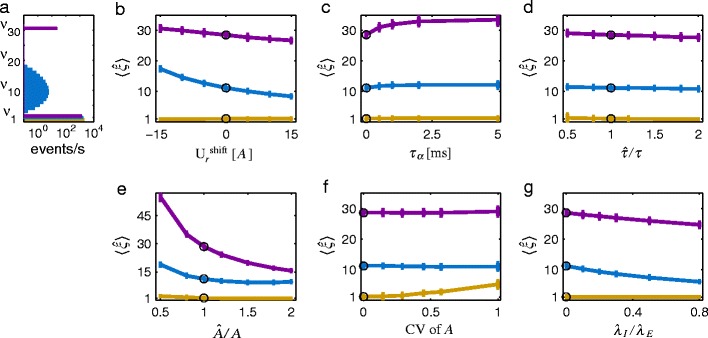
Impact of misestimated kernel (*parameters*) on mean estimated maximal order of correlation. Three surrogate data sets have been analyzed. *Purple*: $N=1000, N_{c}=1000, c=0.01, T=100 s, \lambda =2 spikes/s, \tau =10$ ms. *Yellow*: As purple but with $c=0$. *Blue*: $N=200, N_{c}=200, c=0.01, T=100s, \lambda =10 spikes/s, \tau =10$ ms. The spike trains have been filtered with an exponential kernel and the resulting signal has been analyzed with an exponential kernel with true (or mean in **e**, respectively) amplitude *a* and time constant $\tau $ unless stated otherwise. $\hat {\xi }$ has been averaged over 50 data sets per parameter setting. *Error bars* denote one time standard deviation. **a** Correlation structure of the three sample data sets. **b** The signal is considered as subthreshold membrane potential fluctuations where not the actual resting membrane potential $U_{r}=0$ but $U_{r}+U_{r}^{\mathrm {shift}}$ has been subtracted. $U_{r}^{\mathrm {shift}}$ is in units of the amplitude *a* of the filter kernel or postsynaptic potential, respectively. **c** Synaptic current as $\alpha $-synapse with time constant $\tau _{\alpha }$. **d** Time constant $\tau $ misestimated as $\hat {\tau }$. **e** Amplitude A misestimated as $\hat {{A}}$. **f** Presynaptic activity with lognormal distributed amplitude a for various coefficients of variation $\mathrm {CV}$ of the distribution. **g** Presynaptic activity consisting of an excitatory correlated population with spike rate $\lambda _{E}$ per neuron and an additional inhibitory independent population of the same size with spike rate $\lambda _{I}$ per neuron

Employing the CPP as a model for neuronal population activity does not necessarily imply that all single-neuron spike trains obey Poisson statistics (see e.g. Ehm et al. 2007; Staude et al. 2010c). Nevertheless, this scenario represents a case which is typical for applications of the concept, and which is well manageable in stochastic simulations. Since neuronal spiking often deviates from Poisson (see e.g. Averbeck 2009; Davies et al. 2006; Griffith and Horn 1966; Holt et al. 1996; Maimon and Assad 2009; Neubauer et al. 2009; Shinomoto et al. 2009) and postsynaptic spiking is affected by the non-Poissonian characteristics of its spiking input (Câteau and Reyes 2006; Deger et al. 2012; Ly and Tranchina 2009), we have to ask how reliable the estimates by CuBICm for non-Poissonian processes are. To study this we simulated correlated Poisson processes (PP) and non-Poissonian processes with lognormal distributed inter-spike intervals (nonPP) as described previously (Reimer et al. 2012). The resulting correlation structure has a binomial shape for $n\geq 2$ (see blue data set in Fig. 7a). Figure 6 shows the ratio of the inferred maximal order of correlation for non-Poissonian processes and Poisson processes, $\hat {\xi }^{\mathrm {nonPP}}/\hat {\xi }^{\mathrm {PP}}$. We varied the coefficient of variation of the lognormal inter-spike intervals and used different time constants of the filter kernel. For large time constants of the kernel the maximal order of correlation may be over- or underestimated. For instance, the actual $\xi $ is underestimated for $\tau =50\,\mathrm {ms}$ and a lognormal processes with $\mathrm {CV}=1$ which reflects the fact that a lognormal process with $\mathrm {CV}=1$ is not a Poisson process. In contrast, the maximal order of correlation is well estimated for $\mathrm {CV}=2$ and the same $\tau $. For small time constants the inference is, however, hardly impaired for any non-Poissonian process.

### Misestimated parameters and kernel

In order to estimate the maximal order of correlation $\xi $ the kernel and its parameters need to be known. For instance, for an exponential filter kernel, the time constant $\tau $ and the amplitude *a* have to be specified. Therefore, we investigated to which degree errors in the estimation of kernel parameters can impair the test performance of CuBICm. In so doing, we specifically consider the case of subthreshold membrane potential fluctuations.

#### Subtracting the wrong resting membrane potential

If the signal in question is a subthreshold membrane voltage trace, the resting membrane potential $U_{r}$ has to be subtracted before analysis (cf. Section 2.2.2). Figure 7b shows what happens if one falsely uses $U_{r}+U_{r}^{\mathrm {shift}}$ for normalization. An underestimation of the resting membrane potential (i.e., $U_{r}^{\mathrm {shift}}<0$) leads to an overestimation of the actual order of correlation, and *vice versa*. Note, that by assuming $U_{r}+U_{r}^{\mathrm {shift}}$ instead of $U_{r}$ the mean of the signal is changed to $\kappa _{1}[S]-U_{r}^{\mathrm {shift}}$, whereas the second and third cumulant are shift-invariant and remain the same. Hence, the pairwise correlation coefficient (cf. Staude et al. 2010c)

12 $$ c=\left(\frac{N\kappa_{2}{[S]}\int \phi}{\kappa_{1}{[S]}\int \phi^{2}}-N\right)/\big(N_{c}^{2}-N_{c}\big) $$

is underestimated if $U_{r}^{\mathrm {shift}}<0$. The same magnitude of the third cumulant, however, can be realized with weaker pairwise correlations, only if the maximal order of correlation is higher. Thus, CuBICm overestimates the actual $\xi $ for underestimations of the resting membrane potential.

#### Choosing wrong kernel type

So far, we considered processes where the presynaptic spike activity had been filtered with an exponential kernel. That is, if we consider the subthreshold activity *S* as membrane potential fluctuations we assumed pulse-like synaptic current input (cf. Eq. (4)). However, a more realistic description of the postsynaptic potential is to model the synaptic current as an $\alpha $-synapse (Tuckwell 1988; Rotter and Diesmann 1999). The synaptic time constant $\tau _{\alpha }$ determines the rise time of the postsynaptic potential evoked by one spike in the input and for $\tau _{\alpha }\rightarrow 0$ the postsynaptic potential approaches the shape of an exponential kernel. We generated corresponding surrogate data for different values of $\tau _{\alpha }$ while we normalized the amplitude of the postsynaptic potential *A* to one. The maximal order of correlation has, however, been inferred by using an exponential kernel. The membrane time constant $\tau $ and the amplitude of the postsynaptic potential *A* were chosen as the actual values.Figure 7c shows that the application of the wrong kernel impairs the estimate of $\xi $ more for larger synaptic time constants $\tau _{\alpha }$. However, the overall degree of overestimation of $\xi $ is very small.

#### Misestimated time constant $\tau $

For an exponential kernel the maximal third cumulant under $H_{0}^{k}$ does not depend on the time constant $\tau $, as a straightforward calculation shows (cf. Eq. (15)). Hence, for the hypothesis test the time constant does not need to be estimated. However, in order to account for the correlations of the samples, a shot noise process similar to the one under investigation has to be simulated and $\tau $ has to be specified. Figure 7d shows that a value $\hat {\tau }$ different from the true $\tau $ has only a small effect on the test performance of CuBICm. As was mentioned in Section 3.2, the bigger the time constant is the larger is the bias in underestimating the standard deviation of the third sample cumulant. Hence, if the estimated time constant is larger than the true one, due to our correction method we overestimate the standard deviation of the normal distribution under the null hypothesis. Therefore, we underestimate the maximal order of correlation if any correlations are present in the input (blue and purple line in Fig. 7d).

#### Misestimated amplitude *A*

As opposed to the time constant $\tau $ the amplitude *A* of an exponential kernel affects the cumulant $\kappa _{3,k}^{*}$ according to Eq. (15). In line with this, misestimation of *A* has a stronger impact on the estimation of $\xi $ than the same relative bias in estimating $\tau $ (see Fig. 7e). More precisely, an underestimation of *A* can lead to an overestimation of the actual order of correlation and *vice versa*. Intuitively, this can be explained in the following way: Imagine one presynaptic spike elicits a postsynaptic potential with unit amplitude. If, however, we assume wrongly that one presynaptic spike results in a postsynaptic potential with amplitude 0.5, a measured postsynaptic potential of amplitude 1 has to be interpreted as being the result of two coincident spikes. This is actually exactly what we find for the example of 1000 neurons with synchronized presynaptic spike activity of only one order (see Fig. 7e, purple line). For less simple correlation structures the dependence of $\xi $ on $\hat {A}/A$ seems to be less straightforward although qualitatively similar (blue line).

### Fixed filter kernel

In order to infer by CuBICm higher-order correlations from subthreshold activity not only a kernel and its parameters have to be chosen but rather we are restricted to this one fixed kernel. We treat here two of its most serious implications.

#### Unequal synaptic amplitudes

In our previous simulation studies we assumed that each presynaptic spike had the same impact on the membrane potential. This means, in particular, that synaptic inputs at different locations have the same synaptic efficacy at the soma which may (see e.g. Häusser 2001) or may not (see e.g. Williams and Stuart 2002) hold true. We investigated how reliable the estimates of $\xi $ are when there is no dendritic ‘democracy’. In doing so, each input spike train was filtered with a different amplitude *A* of the exponential kernel where *A* was drawn from a lognormal distribution (cf. Lefort et al. 2009). We set the mean of *A* to 1 and varied its variability as measured by the coefficient of variation $\mathrm {CV}$ of the underlying distribution. The resulting shot noise processes were analyzed with $A=1$. We find that for independent presynaptic neurons some degree of variability of *A* can be tolerated and only for very big CV the maximal order of correlation is overestimated (Fig. 7f, yellow line). For our examples with correlated input one can hardly see any effect of non-identical synaptic efficacies on the estimation of $\xi $ (Fig. 7f, blue and purple). We observed that the actual shape of the distribution of *A* does not have a strong impact and similar results are obtained if *A* is uniform or gamma distributed (not shown).

#### Excitatory and inhibitory input

The assumption of a fixed filter kernel implies in particular that we consider either excitatory or inhibitory presynaptic activity. We mimicked the scenario where the excitatory activity could not be well isolated experimentally and some inhibition is still active. More precisely, we generated additionally *N* independent spike trains with rate $\lambda _{I}$ and convolved them (for simplicity) with the same filter kernel as we used for the excitatory activity but with opposite sign. Figure 7g shows that the maximal order of correlation is the more underestimated the larger the ratio of inhibitory spike rate $\lambda _{I}$ and excitatory spike rate $\lambda _{E}$. The decrease/gradient is very small, though. The underestimation of $\xi $ is due to the fact, that the third sample cumulant of *S*, $k_{3}$, and the variance of $k_{3}$ are on average smaller than we assume for them under $\mathrm {H}_{0}^{k}$. These differences increase with both *k* and the firing rate of the inhibitory population as we show in Appendix C.

### Model of layer 5 pyramidal cell

**Fig. 8:**
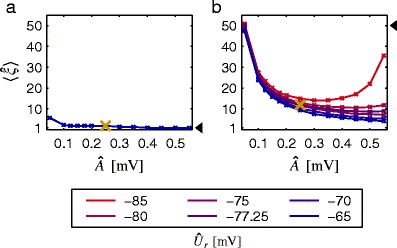
Inferred maximal order of correlation from the membrane potential fluctuations of a multi-compartment model of a reconstructed layer 5 pyramidal cell (Hay et al. 2011). Mean results of five simulations are shown for different estimated EPSP amplitudes $\hat {A}$ and estimated resting membrane potential $\hat {U}_{r}$ (color coded). The model has various active ionic currents where we removed the sodium channels to prevent the neuron from spiking. 10,000 (2,000) conductance based AMPA (GABA) synapses with rise time 0.2 ms (1 ms), decay time 1.7 ms (10 ms) and peak value 3 nS were randomly distributed, where all presynaptic neurons fired 1 (2.5) spikes/s. All spike trains were simulated as Poisson processes, which were independent of each other within the inhibitory population. **a** Uncorrelated excitatory population. **b** 200 neurons of the excitatory population are correlated with a pairwise correlation coefficient *c* = 0.2, and an order of correlation of 50. *Black triangles* mark maximal order of correlation. *Yellow crosses* mark the result which is obtained if $\hat {A}$ and $\hat {U}_{r}$ are closest to the true values. Simulation time was 100 s

Figure 8 shows the results for a simulation where various assumptions of CuBICm are violated at the same time. More precisely, we used a biophysical model of a neocortical layer 5b pyramidal cell from Hay et al. (2011, morphology: Fig. 1, model: Fig. 4 therein) which we obtained from ModelDB (accession number 139653; Hines et al. 2004). To prevent the neuron from spiking, we removed the sodium channels but kept the various other channels similar to a block with TTX. We generated an excitatory presynaptic population of 10,000 Poisson neurons which either fired independently with 1 spike/s (Fig. 8a), or 200 of the neurons showed correlated spiking with a pairwise correlation of 0.2 and exhibited correlations of order 50 (Fig. 8b). Additionally, we simulated an inhibitory independent Poisson population of 2,000 neurons which elicited on average 2.5 spikes/s each. The input spike trains were assigned to randomly placed synapses, where the synaptic integration was simulated using the *Python* interface for the *NEURON* simulation environment (Hines and Carnevale 1997). The conductance transients of the AMPA (GABA) synapses were modeled as difference of two exponentials with rise time 0.2 ms (1 ms), decay time 1.7 ms (10 ms) and peak value 3 nS. Since the EPSP amplitude is state-dependent, and may be reduced during synaptic bombardment (Kuhn et al. 2004), we compared the membrane potential fluctuations for uncorrelated presynaptic spiking with those where each of 2,000 randomly chosen synapses was additionally stimulated with one extra spike. We obtained an average effective EPSP (IPSP) amplitude of 0.24 mV (−0.14 mV), with a coefficient of variation of 0.98 (−0.9).

Simulations were performed for 100 s and for five different realizations of the presynaptic spiking activity. We applied CuBICm to the membrane potential fluctuations by choosing the true membrane time constant of 10 mV. The mean EPSP amplitude was set to values between 0.05 mV and 0.55 mV. Moreover, we analyzed not only data where the true resting membrane potential of −77.25 mV had been subtracted, but we also investigated traces where different $\hat {U}_{r}$ were used. If the presynaptic spiking activity is not correlated, the maximal order of correlation $\xi $ is only slightly overestimated (Fig. 8a). If correlations are present, $\hat {\xi }$ depends more strongly on the assumed mean EPSP amplitude $\hat {A}$ (Fig. 8b). For optimal parameters of $\hat {A}$ and $\hat {U}_{r}$, we obtain $\hat {\xi }\approx 15$. Similar to our findings in Section 5.2.4, $\hat {\xi }$ increases with smaller $\hat {A}$ and it decreases with most of the bigger amplitudes. Subtracting the wrong resting membrane potential has qualitatively the same impact as we revealed in Fig. 7b.

## Discussion

We presented a novel method, CuBICm, to infer higher-order correlations in neuronal network activity from filtered spike activity, in particular, subthreshold membrane potentials. Our method has two major advantages: first, it captures features of the correlation structure of network activity which go way beyond pairwise correlations, and second, it can extract this information from recordings from one cell, thus making multiple electrode recordings and error-prone spike sorting obsolete. Inherent to the nature of intracellular recordings, our approach puts emphasis on the higher-order correlations effectively seen by a single neuron. This is in contrast to previously suggested methods, which were employed to assess the distance dependence of correlations. In this sense, our method constitutes a way to infer the functional aspect of correlations (e.g., how correlations are effectively seen by cells), rather than structural features of neuronal networks or crosstalk between different brain areas (e.g., Ohiorhenuan et al. 2010; Ganmor et al. 2011a). Our new method is applicable to intracellular recordings of short duration, and shows a high sensitivity to weak higher-order correlations.

We devised CuBICm by adaptation of the previously proposed method CuBIC (Staude et al. 2010b, c), which was designed to analyze population spike trains. As a result, CuBICm can provide the same type of information about higher-order correlations, i.e. the maximal order of correlation $\xi $. For population spike trains, another method exists which can reveal even more details on the higher-order correlations: empirical de-Poissonization (EDP; Ehm et al. 2007). The full correlation structure $(\nu _{1},\nu _{2},\ldots ,\nu _{N})$ can be estimated with it. Thus, EDP can highlight differences in the higher-order statistics of two or more populations, which are not reflected in the maximal order of correlation. EDP “extracts $\nu _{n}$ from the characteristic function of the population spike count. Consequently, considering the characteristic function of shot noise processes (cf. Gilbert and Pollak 1960) may be an ansatz to develop a method for membrane potential fluctuations which can estimate the correlation structure $(\nu _{1},\nu _{2},\ldots ,\nu _{N})$ as well.

### Applicability of CuBICm to *in vivo* intracellular recordings

The basic working principle of the family of CuBIC methods is to perform a sequence of statistical hypothesis tests, where the cumulants of measured data with higher-order correlations of unknown order are compared to the cumulants of a model with correlations only up to a certain order included (Staude et al. 2010b, c and Section 2). Apparently, the reliability of such a testing procedure depends on how much the physiological characteristics of the measured system deviate from the assumed model and its parameters. Our model is based on the following set of assumptions: (i) lumped input spike trains are well characterized by a compound Poisson process, (ii) PSPs can be described by a fixed kernel function, (iii) all inputs to the neuron are integrated linearly and, (iv) all inputs are of the same sign, i.e. we have either only excitatory or only inhibitory PSPs. In addition, the resting membrane potential, the membrane time constant and the mean PSP amplitude must be known.

We found that CuBICm is remarkably robust against violations of model assumptions and misestimation of model parameters—even when the recording situation differs in several respects from the underlying model (see Section 5). As Eq. (7) directly shows, a synchronous event of order *n* makes a contribution to the *k*-th cumulant of the membrane potential of order $n^{k}$. Our results suggest that all other perturbations considered here rather affect all cumulants equally, and thus hardly impair the inference of the maximal order of correlation. Here, we discuss the robustness of CuBICm when applying it to *in vivo* intracellular recordings of membrane potentials, specifically with respect to the appropriateness of model assumptions and the estimation of model parameters.

#### Appropriateness of model assumptions

From the above mentioned assumptions, number (iv) appears most problematic. A well-regulated interplay between excitation and inhibition is a generic feature of many known biological networks, and only rarely will a cell in the brain get exclusively either excitatory or inhibitory input. However, as demonstrated in Section 5.3.2, CuBICm is stunningly robust against additional inhibitory input, if higher-order correlations within the pool of excitatory presynaptic neurons are to be measured. To which extent a correlation between excitatory and inhibitory inputs compromises the performance of CuBICm was not tested in our present study. However, such correlations have been experimentally demonstrated in the rat brain (Okun and Lampl 2008; Gentet et al. 2010) and the primate and mouse retina (Cafaro and Rieke 2010). Experimentally, problems associated with inhibition confounding the measurement of HOCs in excitatory inputs can be minimized with the help of pharmacological blockers of GABAergic receptors that act from the cytoplasmic face and can simply be added to the pipette solution (see e.g. Nelson et al. 1994; Lang and Par 1997). Another less invasive method to isolate excitatory input during the measurements would be to clamp the membrane potential to the reversal potential of inhibitory currents using the slow voltage clamp technique (Sutor et al. 2003), which annihilates slow deviations of the membrane potential from the prescribed clamping potential, but does not affect fast deflections like PSPs.

To what extent the assumption (iii) of linearity of PSP summation is justified in cells within an active network is still a matter of debate. Obviously, because of their cable properties (Hodgkin and Rushton 1946; Rall 1959), neurons without specific non-linear mechanisms will integrate in an essentially linear fashion (however, see Kuhn et al. 2004). Indeed, work in acute brain slices has experimentally demonstrated linear integration (Cash and Yuste 1999; Magee and Cook 2000), and even in the intact animal, summation of artificially evoked inputs has been shown to be linear (Léger et al. 2005; Jagadeesh et al. 1993). On the other hand, a rich repertoire of non-linear integration mechanisms has been described in dendritic regions of a number of neuron types, mostly in acute brain slices (Miyakawa et al. 1992; Amitai et al. 1993; Schiller et al. 2000; Larkum et al. 2009), and accordingly non-linear integration properties in single neurons have been described (Nettleton and Spain 2000; Yoshimura et al. 2000). To which extent these mechanisms are actually effective in the intact brain remains a matter of debate until today, and clear evidence for their functional relevance is still lacking. As soon as clear ideas evolve where and when non-linear summation effects play a role, appropriate compensation mechanisms should be included into CuBICm. Given the present uncertainty concerning non-linear summation in the intact brain, it does not seem appropriate to formulate the conditions for such a correction, however. Similarly, sub-threshold, voltage-dependent conductances like $\mathrm {I}_{\mathrm {h}}$ could be taken into consideration for an adapted version of CuBICm, but to date, most quantitative data for channel densities and overall conductances originate from slice work, and it is difficult to estimate their abundance *in vivo*.

While linear summation might be, at present, the most reasonable model, assumption (ii) of all PSPs having the same fixed amplitude and decay time constant does definitely not apply to most biological recordings. Even in a simple cable model of a cell, filtering effects will lead to attenuation and slowdown of PSPs from distant synapses. Indeed, in certain cortical neuron types mechanisms for normalization of amplitude (Magee and Cook 2000) or time course (Williams and Stuart 2000) have been described. However, they seem to be specific to the corresponding cell type or even to certain dendritic regions (Williams and Stuart 2002). In general, PSP amplitudes vary strongly from synapse to synapse, and the distribution of amplitudes at one synapse sometimes shows a heavy tail (Berretta and Jones 1996; Lefort et al. 2009). As was demonstrated in Section 5.3.1, CuBICm works well in similar scenarios. In principle, an adaptation of CuBICm for randomly distributed amplitudes would be feasible. Methodologically this would be similar to the adaptation for non-stationary presynaptic spike activity, which is outlined below. As we also demonstrated in Section 5.2.2, the shape of the PSP does not need to be exactly matched, but an approximation also yields good results.

While the compound Poisson process (i) is a flexible model, it does not capture all features of spiking in biological neurons. We investigated the robustness of CuBICm for non-Poissonian spiking in Section 5.1.2. In fact, our results are in line with our previous findings, where we employed the method empirical de-Poissonization (Ehm et al. 2007; Reimer et al. 2012). It infers higher-order correlations from the population spike count, again assuming the CPP model. We therefore expect that, similarly, the degree of misestimation of $\xi $ by CuBICm does not only depend on the time constant, but also on the detailed spike statistics like firing rate, inter-spike interval distribution, spiking irregularity, and population size. In particular, the results will be most reliable for large populations of sparsely firing neurons—a parameter regime reported for the neocortex (see Barth and Poulet 2012, for a review). Especially in networks which are engaged in the processing of sensory information, spike rates are often strongly fluctuating on a short time scale. The component processes $Y_{n}(t)$ of the compound Poisson process are, however, stationary in the CPP model (see Section 2.1.1), which restricts the type of time variation of spike rates in single neurons. A simple example would be that half of the population spikes only within the first half of the observation interval, while the remaining neurons are silent in this period and fire only in the second half (see Staude et al. 2010b, for less obvious examples). In order to capture also scenarios like rapidly co-fluctuating firing rates of all neurons (Staude et al. 2010b), we extended the CPP model and adjusted CuBIC accordingly. The same approach is also applicable to CuBICm. Briefly, the component processes $Y_{n}(t)$ are conceived as doubly stochastic Poisson processes with a common (but random) rate profile. The cumulants of the population spike count, or postsynaptic subthreshold activity, respectively, are obtained by the law of total cumulance. In doing so, only assuming a parametric family of distributions for the rate fluctuations, and not a specific rate profile, the inference of higher-order correlations for non-stationary processes is made possible.

#### Estimation of model parameters

Among the three parameters which have to be estimated and inserted into the model for proper analysis, the mean PSP amplitude is the most problematic issue. As is shown in Section 5.2.4, an underestimation of the mean PSP amplitude by 50 % can lead to a substantial overestimate of correlation order. The opposite effect occurs for an overestimated PSP size, but it is much less pronounced. This asymmetric dependency of CuBICm suggests that the latter scenario (assumed PSPs bigger than in reality) leads to a conservative use of the method. The order of correlation will in this case, if at all, be slightly underestimated. Estimating PSP amplitudes in individual cells within intact networks is, in fact, not really feasible based on experimental methods available to date. The only reliable measurements of PSP amplitudes come from recordings in acute brain slices, where spontaneous activity is low and individual presynaptic cells can be stimulated repeatedly (either by paired recordings or via light-induced activation). Data from such experiments demonstrate a wide range of amplitudes which strongly depend on age, species, presynaptic/postsynaptic cell type and brain region (for a review see Thomson and Lamy 2007). Most PSP amplitude distributions reported in recent years have their peak below 1 mV, and this number seems to be a good approximation even if inputs from different layers or different cell populations are considered (Schnepel et al. 2011). However, neurons *in vivo* receive the spiking activity of thousands of other neurons which can reduce the PSP amplitude drastically (Kuhn et al. 2004). Thus, estimates should be adapted accordingly (Kumar et al. 2008).

To get a reasonable estimate of the resting membrane potential is, compared to the mean PSP amplitude, much easier in practice, since the only requirement is to block all synaptic inputs for a limited period of time. In the intact animal, this could be achieved either by local application of ion channel or receptor blockers, or, alternatively, by administration of anesthetics which generate pronounced up/down states (Steriade et al. 1993; Mahon et al. 2001). Such pharmacological manipulations can only be performed after the recordings intended for the assessment of higher-order correlations, and the choice of manipulating agent depends on the details of the experimental procedure. In any case, an over- or under-estimation of the resting membrane potential of a few millivolts would not be too detrimental for the estimation of the degree of higher-order correlations, as we demonstrated in Section 5.2.1. However, the resting membrane potential during intracellular recordings can depend on the pipette solution, and drifting offset potentials may lead to a considerably erroneous read-out, so one of the above mentioned pharmacological interventions seem advisable wherever possible.

In general, the PSP decay time constant should (as far as dendritic filtering effects are neglected or covered by working with distributions of time constants rather than a single fixed value) conform to the membrane time constant. This value can, even in *in vivo* intracellular recordings, be easily assessed by brief current pulse injections (Waters and Helmchen 2006; Léger et al. 2005). Moreover, CuBICm is hardly affected by misestimated membrane time constants (see Section 5.2.3).

### Outlook

Recently, intracellular recording techniques have been substantially improved and today allow us to perform single cell recordings from awake freely moving animals (Margrie et al. 2002), or from genetically identified sub-populations of cells (Dittgen et al. 2004). In combination with our new analysis tool, the occurrence of higher-order correlations can now be studied in different activity regimes, brain regions, or populations of neurons. If combined with optogenetic approaches, CuBICm could even help to reveal the contribution of defined groups of cells to correlated activity in neuronal networks.

CuBICm is applicable to any signal which can be conceived (or approximated) as linearly filtered spike activity, and it is not restricted to subthreshold membrane potential fluctuations. For instance, conductance fluctuations could also be analyzed by CuBICm (cf. Rudolph and Destexhe 2006). More recently it has been shown that also the time course of local field potentials in neocortex can be well estimated by convolving a spike train with a linear filter kernel (Rasch et al. 2009) and, thus, suggests an analysis for higher-order correlations by CuBICm.

## Appendix A: Solution of optimization problem

The optimization problem in Section 2.2.1 can be solved via proceeding as in Staude et al. (2010b). The optimum is achieved for $\nu _{n}=0$ for $n=2,\ldots k-1$ and

13 $$ \nu_{1}^{*}=\dfrac{1}{k-1}\left(\dfrac{\xi k_{1}}{\int \phi}-\dfrac{k_{2}}{\int \phi^{2}}\right)$$

14 $$ \nu_{k}^{*}=\dfrac{1}{k(k-1)}\left(\dfrac{k_{2}}{\int \phi^{2}}-\dfrac{k_{1}}{\int \phi}\right)$$

Thus, the $m$-th cumulant maximal cumulant under the null hypothesis $\mathrm {H}^{k}_{0}$ is given as

15 $$\begin{array}{rll} \kappa_{m,k}^{*}&=&\dfrac{\int \phi^{m}}{\int \phi \cdot \int \phi^{2}}\left(k_{2} \int \phi \cdot \left( k^{m-2}+k^{m-3}+\ldots +1\right)\right. \\ && \qquad\qquad\quad\left.  - k_{1}{}\int{}\phi^{2} {\kern-1.5pt}\cdot \left(k^{m-2}{\kern-.8pt}+k^{m-3}{\kern-.8pt}+\ldots+k\right){}\right){\kern-1.5pt}. \end{array}$$

## Appendix B: Variance of third sample cumulant

The variance of third sample cumulant is given by

16 $$\begin{array}{@{}rcl@{}} \mathrm{Var}(k_{3})&=\frac{1}{L}\kappa_{6}&+\frac{9}{L-1}\kappa_{4}\kappa_{2}+\frac{9}{L-1}\kappa_{3}^{2} \\ &&+\frac{6L}{(L-1)(L-2)}\kappa_{2}^{3} \end{array} $$

(Stuart and Ord 1987). Thus, the variance of the third sample cumulant under the null hypothesis, denoted $(\sigma ^{*})^{2}$, is given by plugging-in $\kappa _{m,k}^{*}$ according to Eq. (15) for each $\kappa _{m}$.

## Appendix C: Excitatory and inhibitory presynapticspiking activity

We show here how our simulation results in Section 5.3.2 can be explained theoretically. In doing so, we compare the *m*-th cumulant of the shot noise process *S*, $\kappa _{m}(S)$, and the upper bound for the *m*-th cumulant assumed under $\mathrm {H}_{0}^{k}$, $\kappa _{m,k}^{*}(S)$. The shot noise process *S* can be represented as

17 $$ S=S_{E} + S_{I} $$

where $S_{E}$ ($S_{I}$) is the shot noise process of the excitatory (inhibitory) population.

For the inhibitory spike trains we made the following assumptions:

i) the excitatory and inhibitory spike trains are independent of each other
ii) the *m*-th integral of the inhibitory filter kernel can be decomposed as $\int \phi _{I}^{m}=(-1)^{m} \int \phi _{I_{*}}^{m}$ where $\int \phi _{I_{*}}^{m}>0$
iii) the inhibitory population consists of $N_{I}$ independent Poisson processes with rate $\lambda _{I}$
iv) the firing rate of the excitatory population, $\lambda _{E} N_{E}$, rel- ates to the inhibitory population via $\lambda _{I} N_{I}= q\cdot \lambda _{E} N_{E}$.

18 $$\mathrm{E}\big(\kappa_{m,k}^{*}(S)\big)$$

19 $$=\dfrac{\int \phi_{E}^m}{\int \phi_{E} \cdot \int \phi_{E}^{2}}\left(\kappa_{2}(S) \int \phi_{E} \cdot \big(k^{m-2}+k^{m-3}+\ldots +1\big)-\kappa_{1}(S) \int \phi_{E}^{2} \cdot\big(k^{m-2}+k^{m-3}+\ldots+k\big)\right)$$

20 $$=\dfrac{\int \phi_{E}^m}{\int \phi_{E} \cdot \int \phi_{E}^{2}}\left(\mathrm{E}(k_{2}) \int \phi_{E} \cdot \big(k^{m-2}+k^{m-3}+\ldots +1\big)-\mathrm{E}(k_{1}) \int \phi_{E}^{2} \cdot \big(k^{m-2}+k^{m-3}+\ldots+k\big)\right)$$

21 $$\begin{array}{rll}=\mathrm{E}(\kappa_{m,k}^{*}(S_{E})) &+& \dfrac{\int \phi_{E}^m}{\int \phi_{E} \cdot \int \phi_{E}^{2}}\left(q \kappa_{1}(S_{E}) \int\phi_{I_{*}}^{2}\big(k^{m-2}+k^{m-3}+\ldots +1\big)\right. \\&& \qquad\qquad\quad\quad\; \left.+ \; q \kappa_{1}(S_{E}) \dfrac{\int \phi_{I_{*}}\int \phi_{E}^{2}}{\int \phi_{E}} \big(k^{m-2}+k^{m-3}+\ldots+k\big)\right)\end{array}$$

22 $$=\mathrm{E}(\kappa_{m,k}^{*}(S_{E})) + \underbrace{q \kappa_{1}(S_{E}) \dfrac{\int \phi_{E}^{m}}{\int \phi_{E} } \cdot \left(\dfrac{ \int\phi_{I_{*}}^{2} }{ \int \phi_{E}^{2}}\cdot \big(k^{m-2}+k^{m-3}+\ldots +1\big)+\dfrac{\int \phi_{I_{*}}}{\int \phi_{E}}\big(k^{m-2}+k^{m-3}+\ldots+k\big)\right)}_{b_{2}}$$

Then, the *m*-th cumulant of *S* is given by

23 $$ \kappa_m(S)=\kappa_m(S_E+S_I)$$

24 $$\overset{i)}{=}\kappa_m(S_E)+\kappa_m(S_I)$$

25 $$\overset{ii) + iii) + Eq.\; (7)} {=} \kappa_m(S_E) + \lambda_I N_I \cdot (-1)^m \int\phi_{I_*}^m$$

26 $$\overset{iv)}{=} \kappa_m(S_E) + q \cdot \lambda_E N_E \cdot \int \phi_E \cdot (-1)^m \frac{\int\phi_{I_*}^m}{\int \phi_E}$$

27 $$\overset{Eq.\; (7)}{=} \kappa_m(S_E) + \underbrace{q \cdot \kappa_1(S_E) \cdot (-1)^m \frac{\int\phi_{I_*}^m}{\int \phi_E}}_{b_1} $$

Thus, the actual *m*-th cumulant of *S* deviates from the *m*-th cumulant of the excitatory component $S_{E}$ by $b_{1}$. This amount does not depend on the “fraction” or strength of higher-order correlations, but only on the first cumulant (i.e., population firing rate) of the excitatory population, the relative strength of the inhibitory population firing *q*, and on the respective kernels. In particular, this “bias” is positive for *m* even and negative for *m* odd.

Under $\mathrm {H}_{0}^{k}$ we assume Eq. (15) where we set $\int \phi = \int \phi _{E}$. If the impact of the correlation among samples can be neglected (see however Section 3), the k-statistics are unbiased estimators, i.e. $\mathrm {E}(k_{m})=\kappa _{m}(S)$. Thus, one obtains from Eq. (15) the results in Eq. (22).

Under $H_{0}^{k}$, we assume for the third sample cumulant $k_{3}$ that $k_{3}\sim \mathcal {N}\big (\mathrm {E}\big (\kappa _{3,k}^{*}(S)\big ),\sigma ^{*}\big )$ where $\sigma {*}$ denotes the standard deviation obtained by plugging Eq. (22) into Eq. (16). However, $k_{3}\sim \mathcal {N}(\kappa _{3}(S),\sigma )$ with Eqs. (27) and (16). By comparing $b_{1}$ of Eq. (27) and $b_{2}$ of Eq. (22) one can see, that for *m* odd $\kappa _{m,k}^{*}(S)$ is bigger than $\kappa _{m}(S)$. Yet, the variance is composed of the cumulants up to order 6. For the special case of $\phi =\phi _{E}=\phi _{I_{*}}$, which we considered in Section 5.3.2, we find

28 $$b_{2}=q{} \cdot \kappa_{1}(S_{E}){\kern-.3pt} \cdot{\kern-.7pt} \frac{\int \phi^{m}}{\int \phi} \cdot (2\cdot(k^{m-2}{\kern-.4pt} +k^{m-3}+\ldots +k)+1{\kern-.7pt}).$$

As a result, we find

- $\kappa _{3,k}^{*}(S)$ is bigger than $\kappa _{3}(S)$
- $\sigma {*}$ is bigger than $\sigma $
- the difference increases with *k*.

The null hypothesis $H_{0}^{k}$ has to be rejected if the p-value is smaller than the significance level $\alpha $. $p<\alpha $ is equivalent to $k_{3} >c$ where *c* denotes the $(1-\alpha )$-quantile, i.e. $c=\mathcal {F}^{-1}_{\mathcal {N}\big (\kappa _{3,k}^{*}(S), \sigma ^{*}\big )}(1-\alpha )$. The probability to get a value smaller than *c* for the third sample cumulant is, however, bigger than $1-\alpha $ in our setting:

29 $$\mathcal{F}_{\mathcal{N}\big(\kappa_{3}(S), \sigma\big)}(c)$$

30 $$=\mathcal{F}_{\mathcal{N}(\kappa_{3}(S), \sigma)}\left(\mathcal{F}^{-1}_{\mathcal{N}\left(\kappa_{3,k}^{*}(S), \sigma^{*}\right)}(1-\alpha)\right)$$

31 $$=\mathcal{F}_{\mathcal{N}(\kappa_{3}({\kern-.3pt}S{\kern-.3pt}),\sigma)}\Big({\kern-.5pt}\kappa_{3,k}^{*}{\kern-.5pt}({\kern-.5pt}S{\kern-.5pt}){\kern-1.5pt} +{\kern-2.5pt}\sigma^{*}\sqrt{2} {\rm erf}^{-1}{\kern-1.8pt}(2(1{\kern-1.5pt}-{\kern-1.5pt}\alpha){\kern-1.5pt}-{\kern-2.5pt}1){\kern-1.5pt}\Big)$$

32 $$\geq\mathcal{F}_{\mathcal{N}{\kern-1.5pt}(\kappa_{3}(S), \sigma)}\Big(\kappa_{3}(S){\kern-1.5pt}+{\kern-1.5pt}\sigma\sqrt{2} {\rm erf}^{-1}(2(1-\alpha)-1)\Big)$$

33 $$=1-\alpha $$

Here, erf denotes the error function. From line Eq. (32) to Eq. (33) we made use of the properties a) and b), and the monotonicity of a cumulative distribution function. Thus, the null hypothesis is more frequently “accepted” and, as a result, the actual order of correlation $\xi $ is underestimated.
